# Statin therapy after elective abdominal aortic aneurysm repair improves long-term survival

**DOI:** 10.1093/bjs/znad383

**Published:** 2024-01-10

**Authors:** Fredrik Lilja, Anders Wanhainen, Kevin Mani

**Affiliations:** Department of Surgical Sciences, Uppsala University, Uppsala, Sweden; Department of Surgical Sciences, Uppsala University, Uppsala, Sweden; Department of Perioperative and Surgical Sciences, Umeå University, Umeå, Sweden; Department of Surgical Sciences, Uppsala University, Uppsala, Sweden

## Abstract

**Background:**

Patients with abdominal aortic aneurysms are at high risk of cardiovascular events. Although statin therapy is indicated for most of these patients, no specific recommendation regarding the intensity of therapy exists. The aim of this study was to assess the possible effect of statin therapy on survival of patients undergoing abdominal aortic aneurysm repair and to investigate if high-intensity statin therapy was superior to low–moderate-intensity therapy.

**Methods:**

Data from nationwide Swedish registers on hospital admissions, operations, and medications for patients undergoing elective abdominal aortic aneurysm repair from 2006 to 2018 were collected. The effect of statin use was evaluated in three separate propensity score matched cohorts: perioperative mortality was analysed according to whether patients were on statins before abdominal aortic aneurysm repair or not; long-term survival was assessed according to whether patients were on statins during follow-up or not; and, for those on statins after surgery, long-term survival was analysed according to whether patients were on high-intensity or low–moderate-intensity statin therapy.

**Results:**

Preoperative statin use did not reduce 90-day perioperative mortality (OR 0.99, 95% c.i. 0.77 to 1.28), whilst there was a marked benefit regarding long-term survival for postoperative statin users (HR 1.43, 95% c.i. 1.34 to 1.54). High-intensity statin therapy had no advantage over low–medium-intensity statin therapy with regards to long-term survival (HR 1.00, 95% c.i. 0.80 to 1.25).

**Conclusion:**

In this nationwide propensity score matched cohort study, preoperative statin treatment had no benefit regarding 90-day perioperative survival, but postoperative statin treatment markedly improved long-term survival. No additional benefit regarding high-dose statin treatment could be confirmed in this analysis.

## Teaser

Patients with abdominal aortic aneurysms are at high risk of cardiovascular events. There are no specific recommendations regarding statin therapy for this group. The authors show that postoperative statin use has a marked survival benefit.

## Introduction

The majority of patients undergoing abdominal aortic aneurysm (AAA) repair have concomitant atherosclerotic disease and are at high risk of future cardiovascular events and premature death^1,2^. Treatment with β-Hydroxy β-methylglutaryl-CoA reductase inhibitors (statins) is a cornerstone in the strategy to avoid such events. Therefore, European guidelines on AAA management advocate atherosclerotic risk factor modification including statin therapy if indicated^3^. Statin treatment has been shown in RCTs^4,5^ to significantly decrease perioperative mortality and cardiovascular events after vascular surgery, regardless of co-morbidities, if initiated 4 weeks before surgery and therefore it is strongly recommended^3^.

The American Heart Association (AHA) divides different statin regimens into three groups (low-intensity, moderate-intensity, and high-intensity statin therapy)^6^. However, there is no consensus regarding the optimal statin dose for patients undergoing AAA repair.

No RCTs investigating the impact of statin treatment after AAA repair on long-term outcome or the optimal statin dosage for such therapy have been published. In the absence of RCTs, large population-based observational studies are of value to determine the effect of statin treatment, as a substantial fraction of AAA patients are on this medication. There is a unique possibility to study this topic in Sweden, based on nationwide registers of all patient contacts with specialist care and all dispensed prescriptions, as well as date and cause of death. A unique personal identification number enables cross-linking between these registers.

The downside of inferring treatment effects from observational data is the risk of systematic differences in baseline covariables affecting which patients received treatment in the first place. A way to correct for this bias is propensity score matching, which, by matching patients with a similar probability of receiving treatment, aims to create a treatment group and a control group with similar covariates.

The aim of this nationwide propensity score matched cohort study was three-fold: to assess the effect of statins on perioperative mortality after AAA repair; to assess the effect of statins on long-term mortality and readmission for cardiovascular or cerebrovascular events; and to investigate if high-intensity statin therapy is beneficial in terms of survival and cardiovascular or cerebrovascular events for patients undergoing AAA repair.

## Methods

### Overall study cohort

For this observational, population-based study, all patients undergoing elective index AAA surgery from June 2006 to December 2018 were identified in the Swedish Inpatient Register. Patients were not eligible for inclusion if they had previous AAA surgery, which was checked in the Inpatient Register, dating back to 1998. Patients were identified based on a combination of the ICD^7^ code I71.4 (abdominal aortic aneurysm, intact) and a Nordic Medico-Statistical Committee (NOMESCO)^8^ code indicating a procedure used to treat an AAA (*Supplementary Methods S1*). If a patient had NOMESCO codes for both open repair and endovascular repair, the operation was considered open repair. Date and type of operation, indication for surgery, demographic variables, pre-existing co-morbidities, and postoperative cardiovascular events (defined as re-hospitalization with a cardiovascular event as the main diagnosis) were recorded. Pre-existing co-morbidities were obtained by identifying all hospitalizations since 1998, or contacts with specialized outpatient clinics, for each cohort member before AAA repair and were classified using ICD codes. For each identified patient, all dispensed statin prescriptions were identified in the Prescribed Drug Register^9^. Dispense date, Anatomical Therapeutic Chemical (ATC) number, dose, and number of doses were recorded. From the Cause of Death Register, the eventual date and cause of death were retrieved for every patient.

### Definition of statin use

Patients were considered statin users before surgery either if they had filled enough statin prescriptions to be able to take statins for greater than 80% of the days in the year before surgery or if they had filled at least one prescription of at least 28 pills between 1 and 3 months before AAA surgery to account for initiation of statin therapy before surgery. Similarly, patients were considered statin users after surgery if they had filled enough prescriptions to be able to take statins for greater than 80% of the days after surgery. High-intensity statin therapy was defined, according to the AHA^6^, as treatment with 40–80 mg atorvastatin or 20–40 mg rosuvastatin daily. Any other statin treatment was considered low-intensity or medium-intensity treatment.

### Compared groups

To assess the effect of statin therapy on outcome after AAA repair, three different group comparisons were performed. First, to assess the effect of preoperative statin therapy on perioperative outcome, all primary AAA repairs were assessed by comparing 90-day mortality for patients receiving preoperative statin therapy *versus* patients not receiving preoperative statin therapy. Second, to assess the effect of postoperative statin therapy on long-term outcome, patients who survived 90 days after AAA repair were assessed by comparing those on continuous statin therapy after surgery *versus* those not on continuous statin therapy after surgery. Third, to assess the effect of the intensity of statin therapy, all patients surviving 90 days and who were on continuous statin therapy during follow-up were assessed by comparing patients on high-intensity statin therapy *versus* patients on low–medium-intensity statin therapy.

For each of these analyses, a corresponding cohort of patients were identified, with subsequent propensity score matching between comparative groups before analysis of outcome. Long-term outcome was assessed by comparing overall survival, aneurysm-related survival (defined as free from death from any aortic disease during follow-up or from any cause within 30 days of AAA surgery), and survival free from readmission for cardiac or cerebrovascular events.

A post-hoc analysis of perioperative mortality for patients receiving or not receiving preoperative statin treatment for endovascular aortic repair patients and open repair patients separately was performed by making separate propensity score matched cohorts for endovascular aortic repair patients and open repair patients respectively (*Tables S2*, *S3*).

### Statistical analysis

**Fig. 1:**
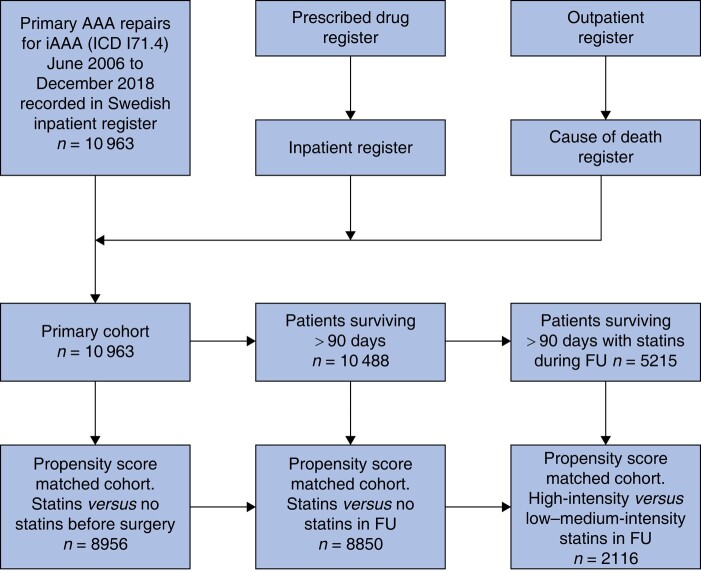
Construction of patient cohorts AAA, abdominal aortic aneurysm; iAAA, intact abdominal aortic aneurysm; FU, follow-up.

The three propensity score matched cohorts (*Fig. 1*) were constructed using 1 : 1 matching with a caliper width of 0.2 of the standard deviation of the logit of the propensity score^10^. Previous knowledge and a directed acyclic graph (DAG) were utilized with R package Dagitty^11^ to select suitable covariates for the propensity score matching (*Fig. S4*)^12^. The covariates selected for propensity score calculation were patient age, year of operation, sex, ischaemic heart disease (IHD), cerebrovascular disease (CVD), chronic kidney disease, diabetes mellitus (DM), hypertension (HT), and chronic obstructive pulmonary disease.

Proportions were compared using chi-squared tests and continuous variables were compared with one-way ANOVA using Python package SciPy^13^. Survival analyses of matched patients were performed using Kaplan–Meier curves and log rank test using Python package Lifelines^14^. Cox regression was used to calculate HRs for overall survival, aorta-related survival, and survival free from readmission for IHD or CVD, whichever occurred first.

### Ethics approval

The study was approved by the Regional Ethics Board of Uppsala (2014/078, 2019–05615).

## Results

Between June 2006 and December 2018, some 11 280 elective AAA repairs were identified. After excluding 317 reoperations, 10 963 index elective AAA repairs remained; 60% (95% c.i. 59% to 61%) of the procedures were performed with endovascular repair. The overall 30-day and 90-day mortality rates were 1.6% (95% c.i. 1.3% to 1.8%) and 2.7% (95% c.i. 2.4% to 3.0%) respectively.

### Preoperative statin use

Before surgery, 59% (95% c.i. 58% to 60%) of patients were on statin therapy. The rate of statin therapy was slightly higher for male patients compared with female patients (60% *versus* 57% respectively, *P* = 0.042). Of the patients on statin therapy, 18% were new users (that is they had filled a prescription of at least 28 pills between 1 and 3 months before surgery as opposed to having filled statin prescriptions earlier before surgery). Of the patients, 2.4% (95% c.i. 2.1% to 2.7%) had additional lipid-lowering medication (bile acid sequestrants, PCSK9 inhibitors, or cholesterol-absorption inhibitors). There was no difference in the 90-day mortality rate between non-statin users, new statin users, and established statin users (2.8%, 2.4%, and 2.8% respectively, *P* = 0.752). Before propensity score matching, patients on statins more often had a history of IHD, DM, HT, and CVD (*Table 1*). After propensity score matching, 8914 patients remained and there was no remaining difference between the groups with respect to covariates (*Table 1*). Before propensity score matching, no difference in 90-day mortality was seen between patients on statin treatment and patients not on statin treatment (OR 0.99, 95% c.i. 0.78 to 1.26). After propensity score matching, 90-day mortality was still similar between statin and non-statin users (OR 0.99, 95% c.i. 0.77 to 1.28).

**Table 1:** Patient characteristics of all included patients

	No preoperative statin treatment	Preoperative statin treatment	P*
**Before propensity score matching**			
Age (years)	73.0 (72.8,73.2)	72.6 (72.5,72.8)	0.011†
Sex, *n*			
Male	3703	5463	0.042
Female	773	1024	
Ischaemic heart disease (%)	9.5 (8.7,10.4)	21 (20,22)	<0.001
Diabetes mellitus (%)	4.6 (4.0,5.2)	8.6 (7.9,9.3)	<0.001
Hypertension (%)	25 (24,27)	34 (32–35)	<0.001
Cerebrovascular disease (%)	2.6 (2.1,3.1)	5.0 (4.5,5.6)	<0.001
Chronic kidney disease (%)	2.4 (2.0,2.9)	3.3 (2.8,3.7)	0.013
Chronic obstructive pulmonary disease (%)	7.8 (7.0,8.5)	7.5 (6.8,8.1)	0.62
**After propensity score matching**			
Age (years)	73.0 (72.8,73.2)	72.1 (72.1,73.1)	0.595†
Sex, *n*			
Male	3700	3741	0.258
Female	772	731	
Ischaemic heart disease (%)	9.6 (8.7,10)	9.5 (8.7,10)	1.000
Diabetes mellitus (%)	4.6 (4.0,5.2)	4.7 (4.1,5.3)	0.920
Hypertension (%)	25 (24.,27)	24 (23,26)	0.328
Cerebrovascular disease (%)	2.6 (2.1,3.1)	2.5 (2.1,3.0)	0.894
Chronic kidney disease (%)	2.4 (2.0,2.9)	2.5 (2.1,3.0)	0.839
Chronic obstructive pulmonary disease (%)	7.7 (6.9,8.5)	7.3 (6.5,8.0)	0.494

Values are proportions (95% c.i.) unless otherwise indicated. *Chi-squared test. †One-way ANOVA.

The post-hoc analysis showed no benefit in terms of perioperative mortality of preoperative statin treatment *versus* no preoperative statin treatment, either in the propensity score matched endovascular aortic repair group (OR 0.95, 95% c.i. 0.59 to 1.51) or in the propensity score matched open repair group (OR 1.16, 95% c.i. 0.74 to 1.82).

### Postoperative statin use

**Fig. 2:**
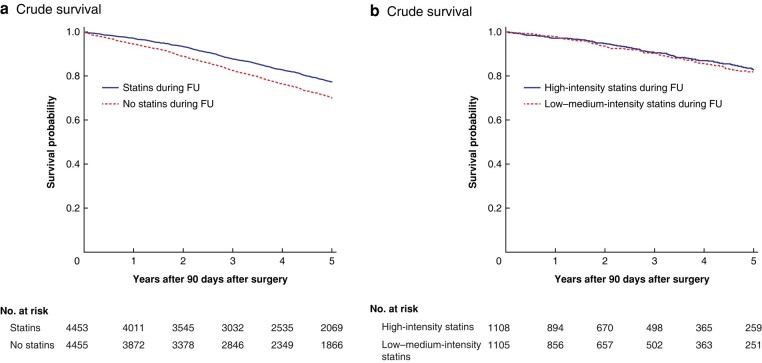
**Long-term survival after abdominal aortic aneurysm repair based on statin therapy. a** Long-term crude survival after abdominal aortic aneurysm repair for patients receiving or not receiving statin therapy during follow-up (*P* < 0.005, log rank test). **b** Long-term crude survival after abdominal aortic aneurysm repair for patients receiving low–moderate-intensity or high-intensity statin therapy during follow-up (*P* = 0.98, log rank test). Events within 90 days after abdominal aortic aneurysm repair are excluded from these analyses of long-term survival. FU, follow-up.

A total of 10 488 patients survived 90 days and 47% (95% c.i. 46.% to 48%) of these were on statin therapy during follow-up. Of the patients, 2.3% (95% c.i. 2.0% to 2.5%) had additional lipid-lowering medication (bile acid sequestrants, proprotein convertase subtilisin/kexin type 9 (PCSK9) inhibitors, or cholesterol-absorption inhibitors) during follow-up. The rate of patients on statin therapy during follow-up was higher for males compared with females (56% *versus* 50% respectively, *P* < 0.001). Before propensity score matching, patients on postoperative statin therapy were slightly younger and more often had a history of DM, HT, IHD, and CVD (*Table 2*). After propensity score matching, 8914 patients remained and there was no remaining difference between the groups with respect to co-morbidities (*Table 2*). The overall long-term survival was better for patients on statins during follow-up (HR 1.44, 95% c.i. 1.34 to 1.54) (*Fig. 2*). Aorta-related survival was higher for patients on statin treatment *versus* patients not on statin treatment during follow-up (HR 1.44, 95% c.i. 1.20 to 1.72), as was survival free from readmission for cardiac or cerebrovascular events (HR 1.36, 95% c.i. 1.27 to 1.46) (*Fig. S5* and *Table 3*).

**Table 2:** Patient characteristics of all patients who survived >90 days after surgery (statin *versus* no statin treatment during follow-up)

	No statin treatment during follow-up	Statin treatment during follow-up	P*
**Before propensity score matching**			
Age (years)	72.9 (72.7,73.1)	72.6 (72.4,72.7)	0.020†
Sex, *n*			
Male	3855	4939	0.067
Female	841	853	
Ischaemic heart disease (%)	9.4 (8.5,10.3)	21 (20,22)	<0.001
Diabetes mellitus (%)	4.5 (3.9,5.1)	8.4 (7.8,9.1)	<0.001
Hypertension (%)	25 (24,27)	33 (32,34)	<0.001
Cerebrovascular disease (%)	2.3 (2.1,3.0)	5.0 (4.4,5.5)	<0.001
Chronic kidney disease (%)	2.2 (1.8,2.7)	3.1 (2.7,3.5)	0.009
Chronic obstructive pulmonary disease (%)	7.7 (6.9,8.5)	7.4 (6.8,8.1)	0.681
**After propensity score matching**			
Age (years)	73.0 (72.8,73.2)	72.8 (72.6,73.0)	0.283†
Sex, *n*			
Male	3698	3736	0.292
Female	759	721	
Ischaemic heart disease (%)	60 (59,61)	61 (59,62)	0.475
Diabetes mellitus (%)	14 (13,15)	14 (13,15)	0.738
Hypertension (%)	5.6 (5.0,6.3)	5.6 (5.0,6.3)	1.000
Cerebrovascular disease (%)	29 (21,30)	28 (27,29)	0.621
Chronic kidney disease (%)	3.5 (2.9,4.0)	3.3 (2.8,3.9)	0.770
Chronic obstructive pulmonary disease (%)	2.8 (2.3,3.3)	2.6 (2.1,3.1)	0.558

Values are proportions (95% c.i.) unless otherwise indicated. *Chi-squared test. †One-way ANOVA.

**Table 3:** HRs for survival in propensity score matched cohorts

	Statins *versus* no statins during follow-up, patients surviving 90 days	High-intensity *versus* low–moderate-intensity statin therapy during follow-up, patients surviving 90 days and on continuous statin treatment
Overall survival	1.44 (1.34,1.54)	1.00 (0.80,1.25)
Aorta-related survival	1.44 (1.20,1.72)	0.84 (0.50,1.44)
Survival free from readmission for ischaemic heart disease or cerebrovascular disease	1.36 (1.27,1.46)	0.81 (0.66,1.00)

Values are HR (95% c.i.).

### High-intensity *versus* low–medium-intensity statin therapy

A total of 5792 patients survived more than 90 days and had continuous statin therapy during FU. After excluding 577 patients on mixed-intensity statin treatment, 5215 patients on either high-intensity statin treatment (21.4%) or low–medium-intensity statin treatment remained. Before propensity score matching, the age and the prevalences of IHD, DM, HT, and CVD were significantly higher among patients on high-intensity statin therapy (*Table 4*). After propensity score matching, 2216 patients remained and there were still higher prevalences of IHD and HT among patients on high-intensity statin therapy (*Table 4*). There was no difference between patients on high-intensity statin therapy *versus* patients on low–medium-intensity statin therapy regarding overall survival (HR 1.00, 95% c.i. 0.80 to 1.25) (*Fig. 2*) or aneurysm-related survival (HR 0.84, 95% c.i. 0.50 to 1.44). Survival free from readmission for cardiac or cerebrovascular events was lower among patients on high-intensity statin treatment, with borderline significance (HR 0.81, 95% c.i. 0.66 to 1.00) (*Fig. S6* and *Table 3*). A sensitivity analysis in which subjects were exact matched on IHD and propensity score matched for the remaining covariables was carried out and resulted in similar results regarding overall survival (HR 1.05, 95% c.i. 0.84 to 1.31), aneurysm-related survival (HR 1.00, 95% c.i. 0.61 to 1.67), and survival free from readmission for cardiac or cerebrovascular events (HR 0.87, 95% c.i. 0.71 to 1.07).

**Table 4:** Patient characteristics of all patients who survived >90 days after surgery (high-intensity *versus* low–medium-intensity statin treatment during follow-up)

	Low–medium-intensity statin therapy	High-intensity statin therapy	P*
**Before propensity score matching**			
Age (years)	72.9 (72.7,73.1)	70.9 (70.5,71.3)	<0.001†
Sex, *n*			
Male	3481	968	0.102
Female	620	146	
Ischaemic heart disease (%)	15 (14,16)	30 (27,32)	<0.001
Diabetes mellitus (%)	7.0 (6.2,7.8)	11 (9.5,13)	<0.001
Hypertension (%)	30 (29,31)	39 (36,42)	<0.001
Cerebrovascular disease (%)	3.9 (3.3,4.5)	6.5 (5.1,8.0)	<0.001
Chronic kidney disease (%)	2.7 (2.2,3.2)	3.1 (2.0,4.1)	0.604
Chronic obstructive pulmonary disease (%)	7.4 (6.6,8.2)	7.7 (6.2,9.3)	0.779
**After propensity score matching**			
Age (years)	71.0 (70.6,71.4)	71.0 (70.6,71.3)	0.794†
Sex, *n*			
Male	979	962	0.303
Female	129	146	
Ischaemic heart disease (%)	24 (22–27)	29 (27,32)	0.008
Diabetes mellitus (%)	8.8 (7.2,11)	11 (9,13)	0.117
Hypertension (%)	33 (30,36)	38 (36,41)	0.009
Cerebrovascular disease (%)	5.6 (4.2,7.0)	6.4 (5.0,7.9)	0.474
Chronic kidney disease (%)	2.1 (1.2,2.9)	3.1 (2.1,4.1)	0.180
Chronic obstructive pulmonary disease (%)	6.3 (4.9,7.8)	7.8 (6.2,9.3)	0.213

Values are proportions (95% c.i.) unless otherwise indicated. *Chi-squared test. †One-way ANOVA.

## Discussion

The main findings of this nationwide propensity score matched cohort study are three-fold: first, preoperative statin therapy appears to have no effect on 90-day perioperative mortality after AAA repair; second, postoperative statin treatment seems to have a significant beneficial effect on long-term survival; and third, high-intensity statin therapy does not seem to contribute to improved survival compared with low–medium-intensity statin therapy. In addition, less than half of the patients who underwent AAA repair in this contemporary Swedish cohort received continuous statin therapy during follow-up.

The observed beneficial effect of postoperative statin therapy on long-term survival is in line with both meta-analyses^15,16^ and a large population-based observational study^17^. Several studies and meta-analyses of them^16,18^ also suggest that preoperative statin treatment has a beneficial effect on perioperative survival, which is in contrast with the results of the present study.

The prevalence of atherosclerotic cardiovascular disease (ASCVD) is high among AAA patients^19^ and therefore it is not surprising that statin therapy in this population is beneficial, as statins have been shown to alter cardiovascular risk, both by mediation of low-density lipoprotein (LDL)^20^ and by mechanisms, not involving serum lipids, that modify endothelial function and plaque stability^21^. It is also well established that primary prevention with statins in a population with cardiovascular risk, regardless of the presence of an AAA, is effective and safe^22^.

There is no obvious explanation for the lack of difference in perioperative mortality among statin and non-statin users in the present study. However, three observations can be made. First, there is heterogeneity regarding the definition of a statin user in papers investigating the effect of preoperative statin therapy on perioperative mortality. Some^17^ define a statin user as a patient taking at least one statin pill during their time in hospital, whilst others^23^ consider a patient to be a statin user if statin use is documented at least once during the 1–3 months before surgery. The present study uses the Swedish Prescribed Drug Register and patients were only considered users if they had their statin prescriptions filled at a pharmacy. Considering the known poor adherence to statin therapy^24^, it is possible that a proportion of the patients classified as statin users in the present study did not take their prescribed medication before surgery, which would then result in an underestimation of treatment effect. Second, the present study uses propensity score matching to adjust for confounding variables, which is only used in two of the studies included in the available meta-analyses^15,16^. O’Donnell *et al*.^17^ used propensity score weighting and showed only a modest, yet statistically significant, survival benefit for statin users. Mathisen and Abdelnoor^25^ showed a more marked benefit, but only for patients undergoing open repair, which contrasts to the present findings, where there was no benefit of preoperative statin use in terms of perioperative mortality, regardless of endovascular or open repair. Third, the discrepancy between the benefit in terms of perioperative mortality in the present study and the two available RCTs could, at least in part, be explained by the inclusion of a mix of vascular surgery patients, about half of which were AAA patients undergoing open repair. This might be interpreted as statin treatment being less beneficial to AAA patients compared with patients with atherosclerotic occlusive disease^4,5^.

The improved long-term aorta-related survival for postoperative statin users could potentially be explained to some extent by the pleiotropic effect on vessel walls. Some studies have suggested that statin therapy may reduce the growth of other untreated aneurysms (that is thoracic aortic aneurysms)^26^. Furthermore, statins have been associated with a lower risk of sac expansion after endovascular repair, which, in turn, is known to increase the risk of aortic complications^27,28^. The present study can, however, not support or reject any association between statin therapy and aneurysm sac behaviour over time; this merits further evaluation in future studies.

To the authors’ knowledge there is no published research regarding the effect of different intensities of statin therapy on long-term survival after AAA repair. Alshaikh *et al*.^29^ found no additional beneficial effect of high-intensity statin therapy on short-term mortality after AAA repair. Although beneficial in terms of readmission due to IHD or CVD, it is somewhat surprising that no reduction in all-cause mortality could be seen, as it has been reported that less LDL is always better when it comes to both overall survival and reduction of cardiovascular events^30^. There are several factors to take into consideration when interpreting these results. First, there are residual confounders that may have an impact on the results of the present study, such as LDL levels, obesity, and smoking. It is possible that the patients on high-intensity statin treatment are worse off in terms of these, and potentially other unknown factors, and therefore may be at higher risk of mortality. Second, during the time of the study, the presence of an AAA or AAA repair was not considered an indication for high-intensity statin therapy in the AHA guidelines for secondary prevention^31^. It was not until the 2018 AHA guidelines that high-intensity statin therapy was recommended for all patients with clinical ASCVD including AAA^6^. The scientific support for this recommendation^32–36^ is not based on studies on surgical cohorts and therefore may not be directly translatable to AAA repair patients. Third, the propensity score matching for patients on high-intensity or low–medium-intensity statin therapy does not fully compensate for the higher prevalences of HT and IHD among patients on high-intensity statin therapy, although the sensitivity analysis with exact matching on IHD did not alter the results. Fourth, data from myocardial infarction patients show that many patients do not reach satisfactory LDL levels, despite high-intensity treatment^37^, which would likely have an impact on the results. Because data on LDL levels are lacking in the present study it is not clear if this applies to this study population. An evaluation of LDL levels in AAA patients and their correlation with cardiovascular events and survival would be of value in future studies. To fully evaluate the effect of high-intensity statin therapy after AAA repair an RCT would be required.

The relatively small fraction of AAA repair patients on statin therapy is in line with previous research, both in AAA patients and among patients with peripheral artery disease^17,38^. The reasons for this are likely a combination of the failure of AAA patients’ physicians to prescribe adequate medical therapy and the previously known poor adherence to statin therapy^24^. One could indeed argue that adherence to statin therapy is a marker for compliance to medical advice and adequate medical care in general and therefore may in part explain the better outcome for patients who take their statins. Against the background of the marked benefit regarding long-term outcome in AAA repair patients and that only half the patients are on statin therapy, efforts to increase the fraction of patients on statin treatment may be beneficial in terms of survival. This is supported by a nationwide cohort study of AAA patients investigating trends in medical cardioprotective treatment, cardiovascular disease, and mortality, which shows that a marked increase in statin use is paralleled by a decrease in both admissions for cardiovascular disease and all-cause mortality^39^.

The reasons for the difference between the sexes regarding statin use is not clear, as the indication for statins are the same, regardless of sex. The phenomenon that women are taking less statins than men, even though the indications are the same, has been observed before^40^ and may, at least in part, be due to sex bias in secondary prevention of atherosclerotic disease^41^. It is not possible, with the present data, to say to what extent this difference between the sexes is the result of physicians failing to describe treatment or of patients not taking their medication as prescribed.

The results of the present study should be considered against a background of several limitations. Although registry data were collected prospectively, the study was conducted retrospectively and patients were not randomized to statin treatment. Data on several known confounders, such as smoking status, LDL levels, measured blood pressure, and BMI, were unavailable. Furthermore, the data on co-morbidities in the used registers are in categorical form and do not include information on the severity of conditions. Because of the retrospective study method, it is also possible that ‘unknown unknown confounders’ exist. The only way to address those would be by performing an RCT.

It is important to keep in mind that the view on how statins can be beneficial to AAA patients has evolved during the study interval. It was not until 2018 that the AHA/American College of Cardiology included aortic aneurysm as an ASCVD and thus as an indication for secondary prevention with statins^6,31^. The European Society of Cardiologists still does not view an AAA as an indication for statin therapy, because of the absence of RCTs showing any benefit^42^. This means that it is not known whether patients were on statin treatment for other reasons or because of having an AAA. The likely consequence of this would be that the patients on statin treatment are worse off in terms of cardiovascular co-morbidity. On the other hand, the relatively low fraction of patients taking statins before and after AAA repair makes it possible to create relatively large, matched patient cohorts with similar distributions of co-morbidities. This makes it possible to draw conclusions about statin treatment and AAA repair.

The strength of the study is the reliability of the data. The registers used are mandatory. Every hospital admission, appointment with specialized care, filled prescription, and death is recorded. Migrations to and from other countries are not recorded. However, considering the age of the patients in the present study, the rate of migration is likely very low.

The DAG, in which potential confounders, both known and known unknown, are identified adds transparency to which covariates are included in propensity score matching. Additional transparency is added by the possibility of examining the characteristics of the matched cohorts.

## Supplementary Material

znad383_Supplementary_Data

## Data Availability

Data are available for sharing by the authors upon reasonable request and after ethical approval.
