# Supplementary material for: Statin therapy after elective abdominal aortic aneurysm repair improves long-term survival
Source: Br J Surg. 2024 Jan 10;111(1):znad383. doi: 10.1093/bjs/znad383 (PMC10782213; doi:10.1093/bjs/znad383)

**Statin therapy after elective abdominal aortic aneurysm repair improves long-term survival**

Fredrik Lilja^1^, MD, Anders Wanhainen^1,2^, MD PhD, Kevin Mani^1^, MD PhD

^1^Department of Surgical Sciences, Vascular Surgery, Uppsala University, Uppsala, Sweden

^2^ Department of Perioperative and Surgical Sciences, Surgery, Umeå University, Umeå, Sweden

**Corresponding author.** Dr Fredrik Lilja, Department of Surgical Sciences, Section of Vascular Surgery, Uppsala University, 75185 Uppsala, Sweden.

**Supplementary Materials - Index**

| **Supplementary Methods** |  |
| --- | --- |
| Definitions S1 | *page 2* |
| **Supplementary Figures and Tables** |  |
| Table S2 | *page 3* |
| Table S3 | *page 4* |
| Figure S4 | *page 5* |
| Figure S5 | *page 6* |
| Figure S6 | *page 7* |
|  |  |
|  |  |
|  |  |

**Supplementary Methods**

**S1**, definitions

Patients were included in the initial cohort if they had a registered admission in the Swedish Inpatient Register between 2006-06-01 and 2018-12-31 with ICD code I71.4, intact abdominal aortic aneurysm as their main diagnosis and a NOMESCO code suggesting open or endovascular aortic repair.

The NOMESCO codes PDG10, PDG20, PDG21, PGD22, PDG23 and PDG24 were considered open aortic repair procedures and PDQ10 and PDQ21 were considered endovascular aortic repair procedures. All re-do procedure were excluded based on data from the Swedish Inpatient Registry dating back to 1998.

For the selected cohort data from the Swedish Inpatient and Outpatient Registers ranging from January 1994 to December 2018 were collected regarding comorbidities using ICD codes I20-I25 for ischemic heart disease (IHD), I10-I15 for hypertension, I60-I69 for cerebrovascular disease (CVD), E10-E14 for diabetes mellitus (DM), J40-J47 for chronic obstructive pulmonary disease (COPD) and N17-N19 for chronic kidney disease (CKD).

Re-admission for IHD or CVD was defined as a new admission after AAA-repair with ICD codes I20-I25 or I60-I69.

Aortic related death was defined as death within 30 days of AAA-repair or a cause of death registered in the Swedish Mortality Register with ICD codes: I71.3, I71.4, I71.8 or I71.9.

**Supplementary Figures and Tables**

**Table S2**. Patient characteristics of all patients treated with EVAR.

| **Before propensity score matching** | **No pre-op statin treatment** | **Pre-op statin treatment** | **p*** |
| --- | --- | --- | --- |
| Age, years | 74.9 (74.6 - 75.2) | 74.1 (73.9 - 74.3) | <0.001† |
| Sex (M:F) | 2189:447 | 3340:598 | 0.059 |
| Ischemic heart disease, % | 11.1 (9.9 - 12.3) | 22.7 (21.4 - 24.0) | <0.001 |
| Diabetes mellitus, % | 5.8 (4.9 - 6.7) | 9.7 (8.8 - 10.6) | <0.001 |
| Hypertension, % | 27.7 (26.0 - 29.4) | 35.9 (34.4 - 37.4) | <0.001 |
| Cerebrovascular disease, % | 2.9 (2.2 - 3.5) | 5.7 (5.0 - 6.4) | <0.001 |
| Chronic kidney disease, % | 3.2 (2.6 - 3.9) | 4.2 (3.6 - 4.8) | 0.047 |
| Chronic obstructive pulmonary disease, % | 8.8 (7.8 - 9.9) | 9.0 (8.1 - 9.9) | 0.897 |
| **After propensity score matching** | **No pre-op statin treatment** | **Pre-op statin treatment** | **p*** |
| Age, years | 74.8 (74.6 - 75.1) | 74.8 (74.5 - 75.0) | 0.660† |
| Sex (M:F) | 2183:440 | 2206:417 | 0.411 |
| Ischemic heart disease, % | 11.2 (10.0 - 12.4) | 11.0 (9.8 - 12.2) | 0.895 |
| Diabetes mellitus, % | 5.9 (5.0 - 6.8) | 5.8 (4.9 - 6.7) | 1.000 |
| Hypertension, % | 27.8 (26.1 - 29.5) | 27.7 (26.0 - 29.4) | 0.951 |
| Cerebrovascular disease, % | 2.9 (2.3 - 3.5) | 2.7 (2.1 - 3.4) | 0.802 |
| Chronic kidney disease, % | 3.2 (2.6 - 3.9) | 2.9 (2.2 - 3.5) | 0.470 |
| Chronic obstructive pulmonary disease, % | 8.7 (7.7 - 9.8) | 8.5 (7.4 - 9.5) | 0.768 |

Values in parenthesis are 95% confidence intervals. * Chi square test. † One way ANOVA. EVAR, Endovascular aortic repair.

**Table S3**. Patient characteristics of all patients treated with open repair.

| **Before propensity score matching** | **No pre-op statin treatment** | **Pre-op statin treatment** | **p*** |
| --- | --- | --- | --- |
| Age, years | 70.3 (70.0 - 70.6) | 70.4 (70.1 - 70.7) | 0.685† |
| Sex (M:F) | 1514:326 | 2123:426 | 0.406 |
| Ischemic heart disease, % | 7.3 (6.1 - 8.5) | 17.3 (15.8 - 18.8) | <0.001 |
| Diabetes mellitus, % | 2.9 (2.1 - 3.6) | 6.9 (5.9 - 7.9) | <0.001 |
| Hypertension, % | 22.0 (20.1 - 23.9) | 29.8 (28.0 - 31.6) | <0.001 |
| Cerebrovascular disease, % | 2.2 (1.5 - 2.8) | 4.0 (3.2 - 4.7) | 0.001 |
| Chronic kidney disease, % | 1.3 (0.8 - 1.8) | 1.8 (1.3 - 2.3) | 0.237 |
| Chronic obstructive pulmonary disease, % | 6.2 (5.1 - 7.3) | 5.2 (4.3 - 6.0) | 0.168 |
| **After propensity score matching** | **No pre-op statin treatment** | **Pre-op statin treatment** | **p*** |
| Age, years | 70.3 (70.0 - 70.6) | 70.6 (70.3 - 70.9) | 0.180† |
| Sex (M:F) | 1510:326 | 1518:318 | 0.600 |
| Ischemic heart disease, % | 7.3 (6.1 - 8.5) | 7.6 (6.4 - 8.9) | 0.754 |
| Diabetes mellitus, % | 2.9 (2.1 - 3.7) | 2.7 (1.9 - 3.4) | 0.763 |
| Hypertension, % | 21.9 (20.0 - 23.8) | 22.1 (20.2 - 24.0) | 0.936 |
| Cerebrovascular disease, % | 2.2 (1.5 - 2.9) | 1.9 (1.2 - 2.5) | 0.557 |
| Chronic kidney disease, % | 1.3 (0.8 - 1.8) | 1.1 (0.7 - 1.6) | 0.764 |
| Chronic obstructive pulmonary disease, % | 5.8 (4.8 - 6.9) | 5.6 (4.5 - 6.6) | 0.776 |

Values in parenthesis are 95% confidence intervals. * Chi square test. † One way ANOVA.

**Figure S4**, Directed Acyclical Graph (DAG) to select suitable co-variables to base propensity score matching on.


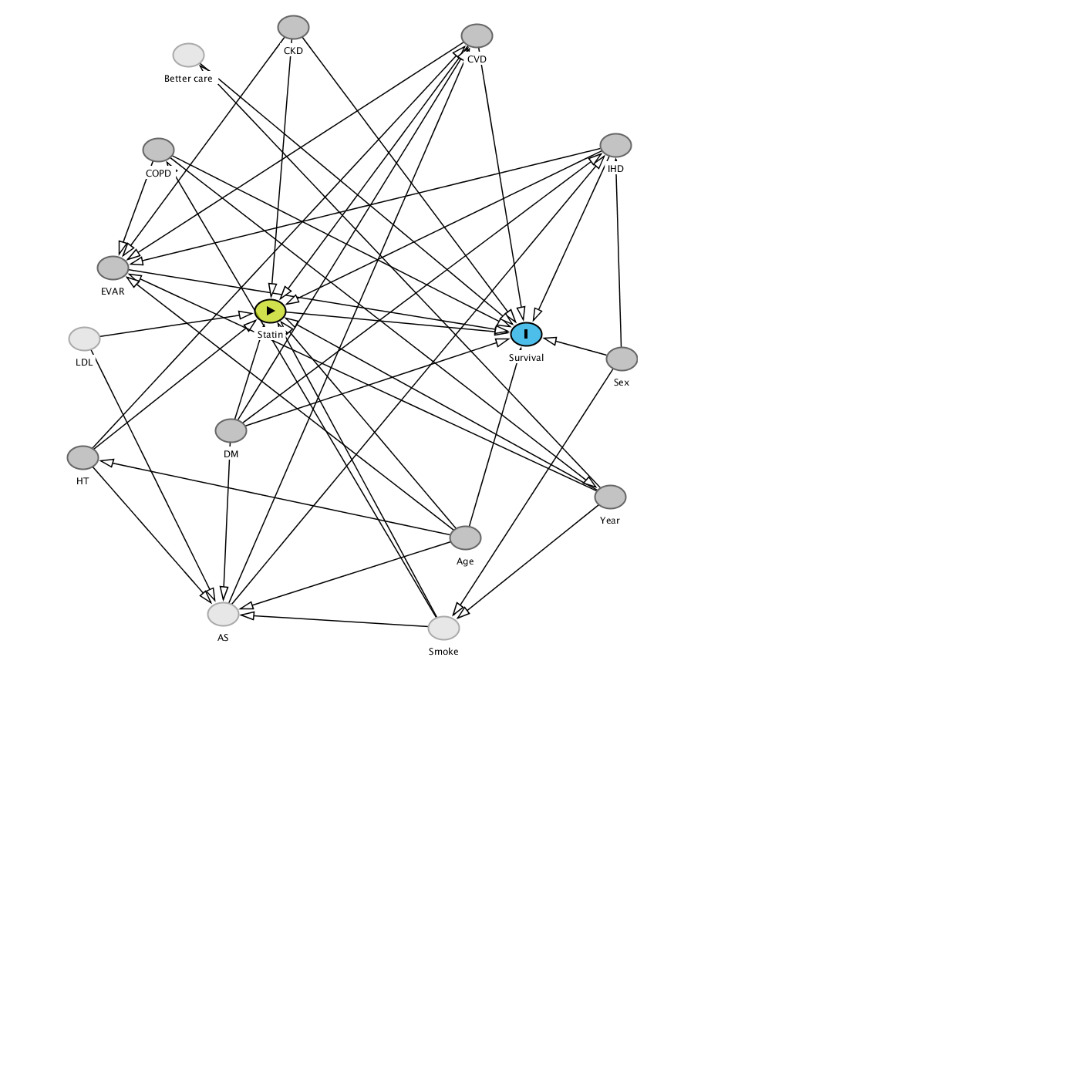


LDL, low-density lipoprotein levels. DM, diabetes mellitus. AS, atherosclerosis. IHD, ischemic heart disease. CVD, cerebrovascular disease, CKD, chronic kidney disease. HT, hypertension, EVAR, endovascular aortic repair. Year, year in which the operation was done. COPD, chronic obstructive pulmonary disease. Better care, assumed improvement in patient care over time. Dark grey nodes represent known variables whereas light grey nodes represent unknown variables.

**Figure S5:** (A) Survival without re-admission for ischemic heart disease or cerebrovascular disease after AAA repair, p<0.005, Log-Rank test. (B) Aneurysm related survival after AAA repair, p<0.005, Log-Rank test. Events within 90-days after AAA repair are excluded from these analyses of long-term survival.


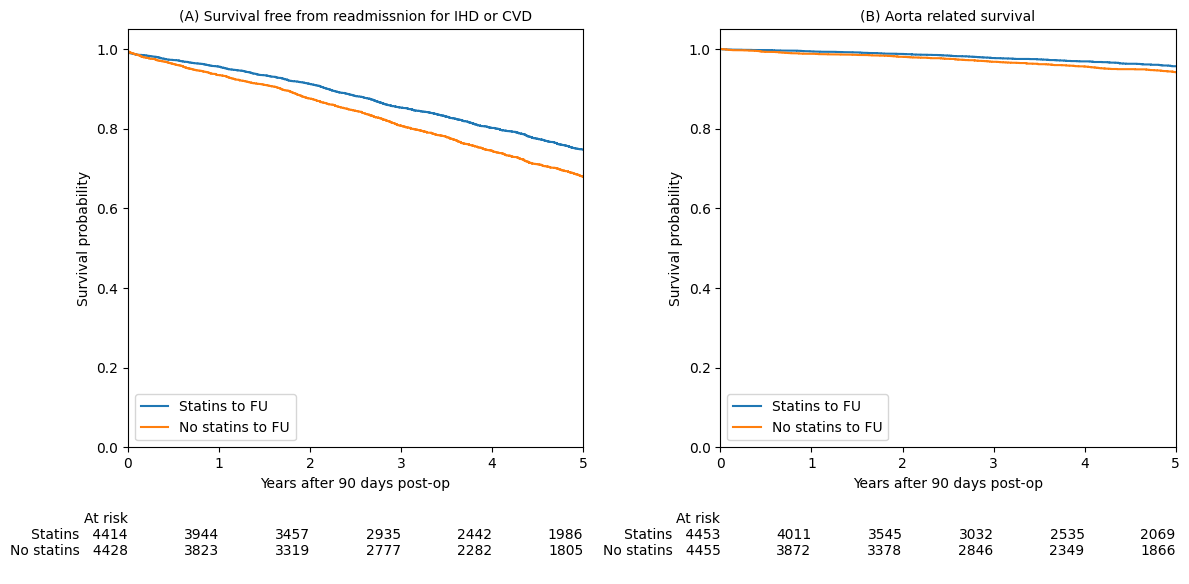


**Figure S6:** (A) Survival without re-admission for ischemic heart disease or cerebrovascular disease after AAA repair, p=0.05, Log-Rank test. (B) Aneurysm related survival after AAA repair, p=0.54, Log-Rank test. Events within 90-days after AAA repair are excluded from these analyses of long-term survival.


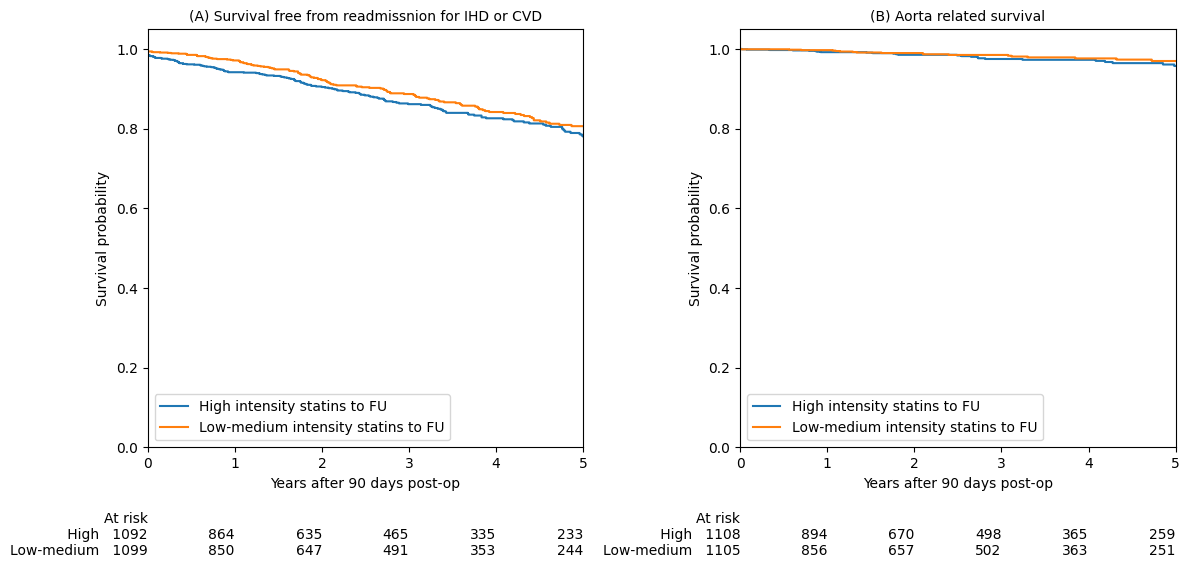

Supplement: znad383_Supplementary_Data [file znad383_supplementary_data.docx]
